# Partial evaluation of fidelity in the implementation of tuberculosis preventive treatment in people living with HIV in Paraguay: an implementation science study

**DOI:** 10.17843/rpmesp.2026.432.15170

**Published:** 2026-06-22

**Authors:** Derlis Duarte-Zoilan, María Patricia Arbeláez Montoya, Dione Benjumea-Bedoya

**Affiliations:** 1 Universidad de Antioquia, Facultad Nacional de Salud Pública, Grupo de Epidemiología, Medellín, Colombia.

**Keywords:** Mycobacterium Tuberculosis, HIV Infections, Coinfections, Implementation Science, Paraguay

## Abstract

**Objectives.:**

To evaluate self-reported adherence to the essential components of tuberculosis preventive treatment (TPT), as an estimate of partial implementation fidelity, and to explore the moderating factors influencing its implementation in two referral services in the Central Department, Paraguay.

**Materials and methods.:**

A descriptive observational mixed-methods study with a convergent parallel design (January-May 2023) was conducted in two referral centers for people living with HIV. Partial implementation fidelity was estimated through self-reported adherence to the content and frequency dimensions of TPT implementation according to the National Tuberculosis Guide. Semi-structured interviews were conducted with key stakeholders to identify barriers and facilitators.

**Results.:**

11/13 physicians and 10 key stakeholders participated. Overall TPT fidelity was low (50.0%). Adherence to content was moderate (64.2%), while adherence to frequency was low (31.3%), evidencing a gap between perceived importance and clinical practice. The awareness/education component showed the lowest performance. The main barriers were identified in the internal environment, including organizational fragmentation between TB/HIV programs, limited resources, absence of operational protocols, and communication deficiencies. At the individual level, erroneous beliefs and low self-efficacy were identified. As facilitators, national normative alignment and the willingness of stakeholders to strengthen implementation were observed.

**Conclusions.:**

Low fidelity reflects mainly organizational and process failures, which highlights the need for facilitation strategies, interprogrammatic articulation, and capacity strengthening to achieve sustained implementation.

## INTRODUCTION

The World Health Organization (WHO) estimates that approximately a quarter of the world's population is infected with *Mycobacterium tuberculosis*. Although not all infected individuals develop the disease or become infectious, about 5-10% may do so, especially those with compromised immune systems ^1^^,^^2^. The COVID-19 pandemic reversed years of progress in the provision of essential tuberculosis (TB) services and in reducing the global burden of the disease. Its most significant impact was an 18% decrease in TB diagnoses and notifications, from 7.1 million in 2019 to 5.8 million in 2020, effectively returning to levels observed in 2012 ^3^^-^^5^. TB continues to be one of the leading causes of death worldwide from a single infectious agent, disproportionately affecting people living with human immunodeficiency virus (HIV) ^6^^-^^9^. TB/HIV coinfection is currently understood as a syndemic, characterized by complex biological and social interactions ^2^.

The international community has reaffirmed its commitment to TB elimination through the WHO End TB Strategy and the 2030 Agenda for Sustainable Development. This approach encompasses multisectoral actions and partnerships at global, regional, cross-border, and national levels ^7^. The strategy prioritizes prevention and research to improve clinical outcomes, with preventive treatment being one of the central pillars of TB prevention ^7^. In the context of TB/HIV coinfection, collaborative activities include treatment and chemoprophylaxis to reduce the risk of progression to active TB among people living with HIV ^9^^-^^11^.

In Paraguay, in 2022, 29,009 people with respiratory symptoms were reported in the Specialized TB System, of whom 87% were evaluated. In the same period, 3,570 new TB cases and relapses were recorded, corresponding to an incidence rate of 43.6 per 100,000 inhabitants, in addition to 380 deaths attributable to TB, with a mortality rate of 5 per 100,000 inhabitants ^12^. The highest concentration of cases was observed in the Central Department and in Asuncion, with 1,500 cases (41.8% of the national total). In relation to TB/HIV coinfection, the highest proportion was recorded in 2020 (8%), with a reduction to 6.8% in 2022 ^12^.

In Latin America and the Caribbean, despite international recommendations, the coverage and effective use of tuberculosis preventive treatment (TPT) in people living with HIV remain suboptimal, with gaps between eligibility and prescription, as well as variability between countries and health services. While collaborative TB/HIV activities have advanced, limitations persist in service integration and in the sustained implementation of TPT, associated with barriers related to health professionals, service organization, and the complexity of clinical algorithms ^13^.

In South America, and particularly in Paraguay, research in implementation science applied to TB/HIV is limited. The available evidence focuses primarily on epidemiological aspects or clinical outcomes, with scarce evaluation of TPT fidelity and of the contextual and organizational determinants that influence its implementation, and with a limited use of formal conceptual frameworks of implementation science ^14^.

In this context, the present study seeks to contribute to closing this knowledge gap. The objective was to evaluate self-reported adherence to the essential components of TPT, as an estimate of partial implementation fidelity, and to explore the moderating factors influencing its implementation in two reference services for people living with HIV in the Central Department, Paraguay, 2023.

**KEY MESSAGES**

Motivation for the study. Advancing implementation science in HIV and tuberculosis is key to reducing the gap between evidence and practice, optimizing the adoption of effective interventions in real-world settings, and improving outcomes in vulnerable populations.

Main findings. The overall fidelity of tuberculosis preventive treatment (TPT) was low, with a gap between adherence to content (64.2%) and frequency (31.3%). Awareness/education showed the worst performance (30.0%). Barriers were concentrated in the internal environment (TB/HIV fragmentation, limited resources, lack of protocols, communication and monitoring deficiencies) and, at the individual level, in erroneous beliefs and low self-efficacy.

Public health implications. Evidence guides strategies to strengthen TPT implementation and reduce community transmission.

## MATERIALS AND METHODS

### Study Design

An observational descriptive study with a parallel convergent mixed-methods design was conducted, aimed at evaluating the implementation fidelity of a preventive intervention in the context of TB/HIV coinfection. The quantitative and qualitative components were collected concomitantly between January and May 2023, analyzed separately, and integrated in the interpretation phase. The quantitative component aimed to quantify the self-reported adherence of medical personnel to the national guideline ^12^, while the qualitative component allowed for the identification of moderating factors that act as barriers or facilitators to implementation. The integration of both approaches was used to explain the observed adherence gaps and generate implications to strengthen the implementation of TB preventive treatment.

### Conceptual framework

The study was guided by two complementary conceptual frameworks of implementation science. First, the Implementation Fidelity Framework by Carroll *et al*. ^15^ was used, which defines fidelity as the degree to which an intervention is implemented as prescribed. According to this framework, it is recognized that implementation fidelity can be influenced by various moderating factors. For their systematic identification and analysis, the Consolidated Framework for Implementation Research (CFIR) ^16^ was used, which allowed for the classification of barriers and facilitators into five domains: intervention characteristics, inner setting, outer setting, characteristics of individuals, and implementation process. The integration of both frameworks allowed for the interpretation of how contextual and organizational factors influence the partial fidelity observed in the content and frequency dimensions of TPT.

### Intervention

The evaluated intervention was TPT in people living with HIV, in accordance with the current national guideline and the clinical algorithm for TB prevention ^12^. TPT was conceptualized as a multicomponent and sequential intervention, whose implementation is conditioned by prior exclusion of active TB. According to the national algorithm, in people living with HIV and in household contacts under five years of age, the performance of latent tuberculosis infection tests is not required (e.g., tuberculin skin test or interferon-gamma release assays) to initiate TPT, provided that active TB has been ruled out. This criterion was explicitly considered in the operational definition of the intervention and in the evaluation of the partial implementation fidelity of TPT in the present study ^12^.

### Logical model

**Figure 1:**
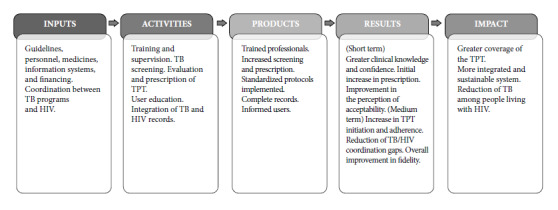
Logical model for the implementation of tuberculosis preventive treatment in people living with HIV. Paraguay, 2023.

TB: tuberculosis; HIV: human immunodeficiency virus; TPT: tuberculosis preventive treatment.

A logical model was developed to describe the causal chain linking the program inputs (training, policy guidelines, and information systems), planned activities, immediate outputs, and the expected short-, medium-, and long-term outcomes of TPT implementation (Figure 1). This model guided the integration and interpretation of the quantitative and qualitative findings.

### Participants, sample, and eligibility criteria

The study was conducted in two reference centers in the Central Department, Paraguay, dedicated to the care of people living with HIV, which function as comprehensive care services within the public health network under the responsibility of the Ministry of Public Health and Social Welfare. These centers concentrate approximately 75% of patients in follow-up. Additionally, managers from the national TB and HIV programs participated.

In the quantitative component, 100% of the physicians responsible for the care of people living with HIV in both centers were invited to participate.

In the qualitative component, participants were recruited by purposeful sampling, and included four program managers (two from TB and two from HIV), two physicians, and four service users. Given the exploratory nature of the study and operational constraints, formal theoretical saturation was not sought.

### Data sources and operationalization of variables

*Quantitative data*

In this study, TPT implementation fidelity was conceptualized as the degree of adherence to the essential components of the national guideline and was partially operationalized, focusing on two dimensions: content (what TPT components are implemented) and frequency (how regularly they are implemented). Other dimensions of the framework, such as population coverage, administered dose, and observed quality of delivery, were not evaluated. These dimensions were measured through medical personnel self-report, using adherence to the national guideline as an operational *proxy* for partial implementation fidelity.

A structured questionnaire was applied that included sociodemographic variables and 40 items organized into three TPT components: diagnosis, counseling-awareness, and clinical management. Each component included items for the content dimension (diagnosis: 10; counseling-awareness: 3; clinical management: 9) and for the implementation frequency dimension (diagnosis: 10; counseling-awareness: 3; clinical management: 5).

Likert-scale responses were dichotomized a priori to define adherence. For the content dimension, adherent responses were considered the categories "important" and "very important"; for the frequency of implementation, the categories "for all new users or annually for follow-up users" and "always for all users".

For each item, adherence was expressed as the percentage of participants with responses classified as adherent. Subsequently, an adherence score per component was calculated as the simple average of the percentages of its constitutive items. Finally, a global adherence score was estimated as the simple average of the three evaluated components, assigning equal weight to each. This value was interpreted as a synthetic estimate of partial implementation fidelity. Adherence levels (and, by extension, partial fidelity) were classified as low (0-50%), moderate (51-79%), and high (80-100%) ^18^^-^^19^.

The questionnaire was adapted from a previously published instrument used to evaluate the implementation of TB screening guidelines in HIV care clinics in Ghana ^17^. The instrument underwent direct translation into Spanish and review by a panel of experts with experience in TB, HIV, and public health, in order to ensure semantic, conceptual, and contextual equivalence. Subsequently, cultural and operational adaptations were made to align it with Paraguay's national tuberculosis guidelines and with the local organization of TB/HIV services. Content validity was established through qualitative consensus of the expert panel, and the instrument was subjected to a pilot test with a small number of professionals, which allowed for minor adjustments in the wording and comprehension of the items. Reliability was evaluated through a descriptive analysis of internal consistency by domains; however, due to the small sample size, it was not possible to estimate robust reliability coefficients, such as Cronbach's alpha.

Two Likert-type scales were used:

Content importance: "not important," "not very important," "neutral," "important," and "very important."

Implementation frequency: "never," "rarely," "for some users depending on symptoms," "for all new users or annually for users under observation," and "always for all users."

*Qualitative data*

Semistructured exploratory interviews were conducted, using guides developed specifically for this study. The guides were formulated based on the CFIR constructs and the moderators of Carroll's implementation fidelity framework, and included open-ended questions aimed at exploring perceptions about the intervention, its complexity, implementation processes, facilitation strategies, and participants' responsiveness. Differentiated versions of the guides were developed according to the type of actor (managers, physicians, and users), maintaining a common conceptual structure and adapting the language and thematic emphases to each participant's role.

### Analysis of the data

*Quantitative analysis*

A descriptive analysis of the study's quantitative variables was performed, including adherence measures by item, adherence scores by component (diagnosis, awareness/education, and clinical management), and the overall estimation of partial implementation fidelity. For each item, adherence was calculated as the percentage of responses classified as adherent relative to the total possible responses. On the Likert-type scale, adherent responses were considered: for the content dimension, the categories "important" and "very important"; and for the frequency dimension, "for all new users or annually for follow-up users" and "always for all users." Subsequently, adherence percentages were estimated by dimensions (content and frequency) and by components, and finally, an overall partial fidelity score was calculated as the proportion of adherent responses relative to the total possible responses in both dimensions. Results were presented with medians and interquartile ranges (IQR) and absolute and relative frequencies. Data processing and analysis was performed using the Epi Info software version 7.2.5.0.

*Qualitative analysis*

The interviews were audio-recorded, literally transcribed, and verified for accuracy. A directed content analysis was performed, initially organizing the transcripts into categories linked to implementation fidelity and, subsequently, applying deductive coding based on CFIR constructs to classify barriers and facilitators across its five domains. The analysis was performed manually using analytical matrices in Microsoft Excel. To strengthen the reliability of the analysis, the coding and interpretation of the findings were reviewed and discussed among the researchers until consensus was reached. The results were subsequently integrated through triangulation with the quantitative findings in the interpretation phase.

*Integration of mixed methods*

Quantitative and qualitative findings were integrated using a convergent triangulation approach, comparing fidelity scores (content, frequency, and integrated component measures) with CFIR-based qualitative results to identify convergences, complementarities, or discrepancies. Interpretation was guided by the logic model, highlighting how program inputs and clinical processes influenced the observed fidelity patterns, and a joint presentation was developed to illustrate the factors explaining implementation gaps.

### Pilot test

A pilot test was conducted with two physicians and two patients to assess the clarity and comprehensibility of the questionnaire and the interview guidelines. The pilot data were not included in the final analysis.

### Ethical Considerations

All data were anonymized and treated confidentially, in accordance with current Paraguayan regulations and the ethical principles of the Declaration of Helsinki. The study was classified as minimal risk, as it did not involve clinical interventions or modification of usual care. All participants provided their written informed consent, and were guaranteed that their participation was voluntary and that their decision would not have repercussions on the care received or on their employment or institutional relationship. The study was approved by the Research Ethics Committee of the Facultad Nacional de Salud Publica de la Universidad de Antioquia (Registration No. 21030002-00231-2022) and by the Direccion General de Vigilancia de la Salud of the Ministerio de Salud Publica y Bienestar Social.

## RESULTS

The quantitative component included 11 physicians, mostly men (63.6%), with a median age of 35 years (IQR: 30-41 years) and a median tenure of 60 months (IQR: 10-144). The majority worked in inpatient care (45.4%) or in a combined inpatient/outpatient consultation modality (36.4%) and resided in the Central Department (90.9%). The qualitative component included 10 participants: six health managers and professionals (TB and HIV programs and physicians), with a median age of 43.5 years (IQR: 42-49 years), and four service users, with a median age of 34.5 years (IQR: 25.5-47 years), with an equitable sex distribution and varied educational levels (table 1).

**Table 1:** Sociodemographic characteristics of participants in the implementation of tuberculosis preventive treatment in people living with HIV. Paraguay, 2023.

Variable			n	%
Physicians-quantitative component (n=11)				
	Gender			
		Male	7	63.6
		Female	4	36.4
	Age (years)^a^		35 (27-45)	
	Work Setting			
		Inpatient	5	45.4
		Outpatient	2	18.2
		Both	4	36.4
	Department of origin			
		Central	10	90.9
		Guaira	1	9.1
	City of origin			
		Asuncion	3	27.2
		Other cities of the Central Department	8	72.8
	Tenure in position (months)^a^		60 (10-144)	
Participants of the qualitative component (n=10)				
	Managers and health professionals (n=6)			
	Age (years)^a^		43.5 (39-55)	
	Gender			
		Male	2	33.3
		Female	4	66.6
	Position			
		TB Program	2	33.3
		HIV Program	2	33.3
		Physician	2	33.3
	Service users (n=4)			
	Age (years)^a^		34.5 (24-52)	
	Gender			
		Male	2	50.0
		Female	2	50.0
	Educational level			
		Primary	1	25.0
		Secondary	1	25.0
		University	2	50.0

TB: tuberculosis; HIV: human immunodeficiency virus.

The percentages were calculated based on the total number of participants in each subgroup.

a Median (interquartile range).

In component 1 (TB diagnosis), moderate adherence to the content was observed, but a low frequency of implementation. Although the importance attributed to the evaluation of signs and symptoms reached 65.6%, the frequency of these practices in routine care was 34.4%. Additionally, a relevant proportion of physicians reported rarely asking about contact with active TB cases and difficulties in accessing diagnostic tests. The integrated adherence of this component was 52.1%, corresponding to a moderate level.

Component 2 (awareness and education) presented the lowest levels of adherence. A considerable proportion of physicians assigned low importance to TPT indication in people living with HIV with a history of contact with active TB, and the frequency of implementing educational activities was scarce. The integrated adherence of this component was 30.3%, reflecting a substantial deficit in the execution of essential educational actions.

**Figure 2:**
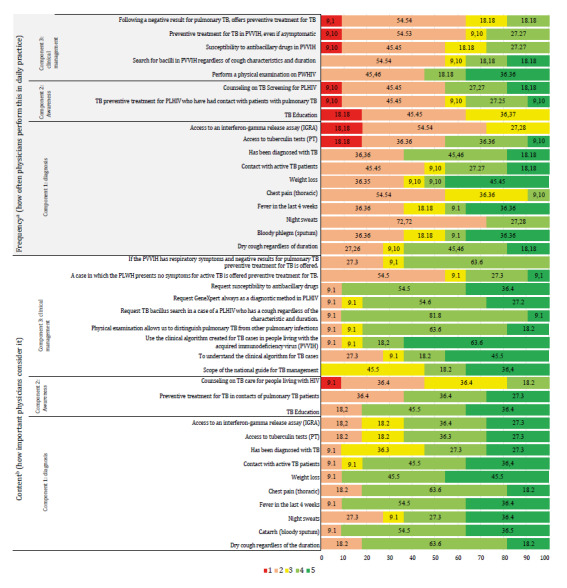
Adherence of physicians in the implementation of tuberculosis preventive treatment in people living with HIV according to dimensions and components. Paraguay, 2023.

^a^ Frequency. 1: never, 2: rarely, 3: for some users depending on symptoms, 4: for all new users or annually for follow-up users and 5: always for all users. ^b^ Content.1: nothing important, 2: little important, 3: neutral, 4: important, and 5: very important.

In component 3 (clinical management), although most physicians recognized the importance of access to clinical algorithms, a gap was evidenced between the knowledge of the guidelines and their practical application. The assessment of clinical content was moderate (67.7%), while the frequency of implementation was low (33.0%), particularly in the indication of TPT for asymptomatic individuals or those with negative results for pulmonary TB. The integrated adherence of this component reached 55.1%, corresponding to a moderate level (figure 2). When integrating the three components, the global level of partial fidelity of TPT implementation —based on the dimensions of content and frequency— was 50.0%, which indicates a low level of operational compliance with national guidelines (table 2).

**Table 2:** Physician fidelity in the implementation of tuberculosis preventive treatment in people living with HIV according to dimensions and components. Paraguay, 2023.

Result of implementation	Dimensions	Components	Responses in the expected categories^a^	Level reached^b^	%
			Responses/ Possible answers		
Adherence	Content	Diagnosis of TB	84/128	Moderate	65.6
		Tuberculosis Awareness	16/33	Under	46.5
		Clinical Management of TB	67/99	Moderate	67.7
	Frequency	Total score	167/260	Moderate	64.2
		Diagnosis of TB	40/110	Low	34.4
		TB Awareness	4/33	Under	12.1
		Clinical management of TB	18/55	Low	33
		Total score	62/198	Low	31.3
Global fidelity	Content and Integrated Frequency	Diagnosis of TB	124/238	Moderate	52.1
		TB Awareness	20/66	Under	30.3
		Clinical Management of TB	85/154	Moderate	55.1
		Total score	229/458	Under	50

TB: tuberculosis.

a Responses in the expected categories correspond to the responses considered adherent for each dimension.

b Fidelity levels were classified as low (0-50%), moderate (51-79%), and high (80-100%).

During the qualitative phase, semi-structured interviews were conducted with four program managers, two physicians from the Comprehensive Care Services, and four users, who provided complementary perspectives on programmatic management, clinical care, and the experience of TB preventive treatment. Qualitative analysis made it possible to identify barriers and facilitators organized into the five CFIR domains. The findings showed that organizational, individual, and contextual factors influence the prescription, initiation, and continuity of preventive treatment in people living with HIV.

The main barriers were concentrated in the internal environment, highlighting the fragmentation of TB/HIV services, the limited availability of organizational resources, knowledge and belief gaps among health professionals, and the absence of systematic planning and feedback processes. Among the facilitators, the staff's willingness to receive training, the interest of some users in accessing information about preventive treatment, and the alignment of national policies with international recommendations were identified. The synthesis of the CFIR constructs, along with the qualitative evidence and its analytical interpretation, is presented in table 3.

**Table 3:** Potential moderators of the fidelity of implementation of tuberculosis preventive treatment in people living with HIV according to CFIR domains. Paraguay, 2023.

Construct ^a^		Evidence (Verbatim fragments from the study)^b^	Interpretation (How does it influence fidelity?)
Domain 1. Intervention			
	Complexity of the intervention	“The programs are completely independent in their management... communication between services is hindered...”; “Limited integration of tuberculosis/human immunodeficiency virus care...”	The intervention requires coordination at multiple levels (TB/HIV), which increases its complexity and reduces fidelity in diagnosis, prescription, and follow-up.
	Quality and robustness of the evidence	“There are widespread doubts about the effectiveness of preventive treatment…”; “Some physicians believe that preventive treatment can cause resistance…”	Evidence perceived as insufficient generates low confidence in preventive treatment, which reduces its adoption and adherence.
	Adaptability	“Follow-up in people living with human immunodeficiency virus is not complex; preventive treatment only needs to be specified and become clearer…”	There are opportunities for local adaptation; however, the essential components are not clearly defined.
	Clarity of the design	“The national guideline does not specify for whom preventive treatment is indicated... it is not clear...”	Lack of clarity in clinical guidelines reduces standardization and increases variability in practice.
Domain 2. External environment			
	Needs and resources of patients	“This is truly the first time I've heard... I had never heard that a treatment existed to prevent tuberculosis...”	Patients do not perceive tuberculosis as a priority risk, which generates passive demand and less pressure for the prescription of preventive treatment.
	External interorganizational networks	“There is no interconnection between the information systems of both programs…”	Fragmented tuberculosis/human immunodeficiency virus systems hinder continuous clinical monitoring and compromise the completeness of implementation.
	Policies and external incentives	National guidelines aligned with the World Health Organization (field observations).	National policies are favorable; however, local implementation is fragile due to low operational articulation.
Domain 3. Internal environment			
	Organizational structure	“Coordination between the regional, district, and local levels is limited…”; “The HIV referral center does not have a tuberculosis program…”	The fragmented organizational structure affects the adequate transfer of the preventive treatment protocol and reduces organizational coherence.
	Available resources	“There are gaps in information recording…”; “Lack of clear normative documents…”	Insufficient technical, informational, and support resources constitute a barrier for consistent implementation.
	Implementation Climate	“It was observed that active tuberculosis case finding was not fully implemented…”	The organizational climate is not favorable; preventive treatment is not prioritized among routine activities.
	Networks of communication and coordination	“The lack of communication hinders collaborative work…”	Poor interprogrammatic communication reduces the quality of implementation.
Domain 4. Characteristics of individuals			
	Knowledge and beliefs about the intervention	“Many colleagues believe that preventive treatment is only for children…”	Incorrect beliefs constitute a critical barrier to adherence in diagnosis and clinical management.
	Self-efficacy	Doubts about resistance, complexity, and lack of training (“we need more information...”)	The low confidence reduces the likelihood of prescription of preventive treatment and the use of the clinical algorithm.
	Stage of change / individual preparation	Interest in training (field diary findings).	There is a willingness for improvement if adequate tools are provided.
	Patients' Needs	“I had heard once... but I never asked and the doctor did not explain anything to me...”	Low patient knowledge limits adherence and acceptance, reducing pressure for implementation.
Domain 5. Implementation process			
	Planning	Absence of clear specific protocols and flowcharts for preventive treatment in adults.	The lack of detailed planning generates inconsistencies in prescription and follow-up.
	Execution	“The active search for tuberculosis cases is not monitored…”	Incomplete execution of the essential components of preventive treatment.
	Participation of stakeholders	Users: “requesting information to receive preventive treatment…"; national programs willing to offer training.	Although there are motivated actors, an articulated implementation mechanism is lacking.
	Reflection and evaluation (feedback)	There is no evidence of regular internal monitoring.	The absence of structured feedback prevents continuous improvement and reduces fidelity.

TB: tuberculosis; HIV: human immunodeficiency virus; TPT: tuberculosis preventive treatment.

a Constructs of the Consolidated Model for Implementation Research (CFIR).

b Textual fragments anonymized from interviews and field observations.

The integration of quantitative and qualitative findings showed concordance between the components with lower implementation fidelity and the domains that concentrated the main barriers. In particular, weaknesses in organizational processes and in the coordination between TB and HIV programs help to explain the low frequency of implementation observed in the evaluated components. A summary of this triangulation is presented in table 4, where the quantitative results, qualitative barriers, and their integrated interpretation are related.

**Table 4:** Triangulation of quantitative and qualitative findings through a logical model in the implementation of tuberculosis preventive treatment in people living with HIV. Paraguay, 2023.

Quantitative Finding	Qualitative finding	Joint interpretation (logical model)^a^
Low frequency of key clinical activities (diagnosis, education, and clinical management).	Internal environment: resource scarcity, absence of clear protocols, inexistence of supervision and feedback.	The clinical processes of preventive treatment are not fully implemented due to weaknesses in inputs and organizational processes.
Health education presented the lowest performance (12.1% compliance).	Individuals: misconceptions of the team, low self-efficacy; users with limited knowledge about tuberculosis preventive treatment.	Insufficient health education and incorrect perceptions about preventive treatment negatively influence adherence to this component.
Low global adherence (50.0%).	Intervention and internal environment: TB/HIV complexity, organizational fragmentation, and limited communication.	Poor integration between tuberculosis and HIV programs interrupts clinical flows and reduces overall fidelity.
Gaps between perceived importance and actual performance.	Process: absence of planning, monitoring, and structured feedback.	Although the team knows the guidelines, the processes are not structured to ensure sustained implementation.

TB: tuberculosis, HIV: human immunodeficiency virus, TPT: tuberculosis preventive treatment.

a Interpretation based on the logical implementation model that integrates quantitative and qualitative results.

## DISCUSSION

The present study evaluated the partial implementation fidelity of TPT in people living with HIV in two reference centers in Paraguay, from an implementation science approach. The main finding was a low overall level of partial fidelity (50%), characterized by a gap between adherence to content (64.2%) and frequency of implementation (31.3%). This pattern suggests that, although professionals recognize the importance of clinical recommendations, their systematic application in clinical practice is limited.

These results are consistent with the implementation science literature, which indicates that knowledge of guidelines, on its own, is insufficient to ensure faithful implementation, especially in complex and multicomponent interventions developed in fragmented healthcare systems ^14^^,^^16^^,^^20^. In particular, the awareness and education component showed the lowest levels of performance, with the lowest implementation frequency (12.1%) and integrated adherence (30.3%), which is concerning given the central role of patient education in promoting the acceptance, adherence, and continuity of TPT ^21^.

The qualitative findings allowed explaining these quantitative patterns. From the perspective of the Consolidated Framework for Implementation Research, barriers were identified in the five domains, highlighting the internal environment as the main focus of obstacles. The organizational fragmentation between TB and HIV programs, limitations in information systems, weak communication channels, and the absence of clear operational guidelines restricted the consistent provision of TPT. These results align with studies conducted in other low- and middle-income contexts, where the limited integration of TB/HIV services compromises the effectiveness of preventive interventions ^22^^-^^23^.

The complexity of the intervention emerged as a key factor. Although national and international guidelines recommend TPT in people living with HIV after ruling out active TB, without requiring additional diagnostic tests ^1^^,^^12^, a significant proportion of physicians attributed low relevance to its indication in asymptomatic adults. This finding reflects how perceived ambiguity and heterogeneous interpretation of clinical recommendations can weaken implementation fidelity, even when policies are aligned with WHO recommendations ^4^^,^^7^. Similar results have been described in studies from Ghana and Peru, where uncertainty about eligibility criteria and concerns about drug resistance reduced healthcare professionals' adherence to TPT ^17^^,^^23^.

Likewise, factors related to the characteristics of individuals negatively influenced fidelity. Erroneous beliefs about TPT, along with lower perceived self-efficacy, affected clinical decision-making and the provision of associated education. Strict monitoring by healthcare professionals is necessary to ensure treatment adherence in people with TB/HIV and improve therapeutic success rates, considering that previous evidence indicates that the trust and perceived competence of professionals constitute key determinants for successful implementation, especially in preventive interventions whose benefits are not immediate ^24^.

Finally, the integration of quantitative and qualitative findings through the logical model and the Conceptual Framework of Fidelity allowed to clarify how deficiencies in inputs (training, regulatory clarity, and information systems) and in processes (screening, education, prescription, documentation, and follow-up) translated into a low frequency of implementation. From a programmatic perspective, these results suggest that improving the fidelity of TPT in Paraguay requires going beyond the dissemination of guidelines and moving towards active facilitation strategies, such as simplified clinical algorithms, continuous training, strengthening of documentation, and systematic audit and feedback mechanisms, which have been shown to improve implementation in other contexts ^25^^-^^26^.

This study presents some limitations. The validation of the instrument was based primarily on expert review and contextual adaptation, without estimation of formal quantitative metrics of validity or reliability, which constitutes a methodological limitation. Secondly, implementation fidelity was partially evaluated, limited to the dimensions of content and frequency and based on self-report by medical personnel, which may have introduced recall and social desirability biases. Furthermore, it was not possible to evaluate other central components of fidelity, such as population coverage, administered dose, or quality of delivery, due to the limited availability of clinical records and access restrictions, which prevented triangulation with objective measures.

Furthermore, the qualitative component was exploratory, with a small sample size and without formal theoretical saturation search, so its findings should be interpreted as indicative and not generalizable. The study was conducted in two specialized reference services, so the results are mainly applicable to similar contexts of HIV care and not necessarily representative of the national level. Finally, although the use of mixed methods strengthened the interpretation of the findings, the observational and cross-sectional design of the study does not allow establishing causal relationships nor evaluating longitudinal changes in TPT implementation.

In conclusion, self-reported adherence to the essential components of TB preventive treatment was low, reflecting limited partial fidelity in the dimensions of content and frequency. The identified moderating factors were mainly concentrated on the organizational environment and on individual characteristics of healthcare professionals, which helps to explain the observed gaps between the importance attributed to the guidelines and their application in clinical practice. These findings suggest that strengthening implementation facilitation, through clear algorithms, continuous training, integration of TB/HIV services, and systematic feedback mechanisms, could improve the implementation of TPT in people living with HIV.
