# Supplementary material for: Partial evaluation of fidelity in the implementation of tuberculosis preventive treatment in people living with HIV in Paraguay: an implementation science study
Source: Rev Peru Med Exp Salud Publica. 2026 Jun 22;43(2):160–70. doi: 10.17843/rpmesp.2026.432.15170 (PMC13456928; doi:10.17843/rpmesp.2026.432.15170)
Supplement: Translation

# Translation: Evaluación parcial de la fidelidad en la implementación del tratamiento preventivo de tuberculosis en personas que viven con VIH en Paraguay: un estudio de ciencia de implementación

**Authors:** Derlis Duarte-Zoilan, María Patricia Arbeláez Montoya, Dione Benjumea-Bedoya

**Affiliations:** 1 Universidad de Antioquia, Facultad Nacional de Salud Pública, Grupo de Epidemiología, Medellín, Colombia.

**Keywords:** Mycobacterium tuberculosis, Infecciones por el VIH, Coinfección, Ciencia de la implementación, Paraguay

## Abstract

**Objetivos.:**

Evaluar la adherencia autoinformada a los componentes esenciales del tratamiento preventivo de la tuberculosis (TPT), como estimación de fidelidad parcial de implementación, y explorar los factores moderadores que influyen en su implementación en dos servicios de referencia del Departamento Central, Paraguay.

**Materiales y métodos.:**

Estudio observacional descriptivo de métodos mixtos con diseño convergente paralelo (enero-mayo de 2023) en dos centros de referencia para personas que viven con VIH. La fidelidad parcial de implementación se estimó mediante la adherencia autoinformada a las dimensiones de contenido y frecuencia de implementación del TPT según la Guía Nacional de Tuberculosis. Se realizaron entrevistas semiestructuradas a actores clave para identificar barreras y facilitadores.

**Resultados.:**

Participaron 11/13 médicos y 10 actores clave. La fidelidad global del TPT fue baja (50,0%). La adherencia al contenido fue moderada (64,2%), mientras que la adherencia a la frecuencia fue baja (31,3%), evidenciando una brecha entre la importancia percibida y la práctica clínica. El componente de sensibilización/educación presentó el menor desempeño. Las principales barreras se ubicaron en el entorno interno, incluyendo fragmentación organizacional entre los programas de TB/VIH, recursos limitados, ausencia de protocolos operativos y deficiencias en comunicación. A nivel individual, se identificaron creencias erróneas y baja autoeficacia. Como facilitadores, se observó alineamiento normativo nacional y disposición de los actores para fortalecer la implementación.

**Conclusiones.:**

La baja fidelidad refleja fallas principalmente organizacionales y de proceso, lo que resalta la necesidad de estrategias de facilitación, articulación interprogramática y fortalecimiento de capacidades para lograr una implementación sostenida.

## INTRODUCCIÓN

La Organización Mundial de la Salud (OMS) estima que aproximadamente una cuarta parte de la población mundial está infectada por *Mycobacterium tuberculosis*. Aunque no todos los individuos infectados desarrollan la enfermedad ni se vuelven infecciosos, alrededor del 5-10% puede hacerlo, especialmente aquellos con compromiso del sistema inmunológico ^1^^,^^2^. La pandemia de COVID-19 revirtió años de progreso en la provisión de servicios esenciales de tuberculosis (TB) y en la reducción de la carga global de la enfermedad. Su impacto más significativo fue una disminución del 18% en los diagnósticos y notificaciones de TB, de 7,1 millones en 2019 a 5,8 millones en 2020, retornando efectivamente a los niveles observados en 2012 ^3^^-^^5^. La TB continúa siendo una de las principales causas de muerte en el mundo por un único agente infeccioso, afectando de manera desproporcionada a las personas que viven con el virus de la inmunodeficiencia humana (VIH) ^6^^-^^9^. La coinfección TB/VIH se entiende actualmente como una sindemia, caracterizada por interacciones biológicas y sociales complejas ^2^.

La comunidad internacional ha reafirmado su compromiso con la eliminación de la TB mediante la Estrategia Fin de la TB de la OMS y la Agenda 2030 para el Desarrollo Sostenible. Este enfoque abarca acciones multisectoriales y alianzas a nivel global, regional, transfronterizo y nacional ^7^. La estrategia prioriza la prevención y la investigación para mejorar los desenlaces clínicos, siendo el tratamiento preventivo uno de los pilares centrales de la prevención de la TB ^7^. En el contexto de la coinfección TB/VIH, las actividades colaborativas incluyen el tratamiento y la quimioprofilaxis para reducir el riesgo de progresión a TB activa entre las personas que viven con VIH ^9^^-^^11^.

En Paraguay, en 2022, se notificaron 29 009 personas con síntomas respiratorios en el Sistema Especializado de TB, de las cuales el 87% fue evaluado. En el mismo período, se registraron 3570 nuevos casos de TB y recaídas, lo que corresponde a una tasa de incidencia de 43,6 por 100 000 habitantes, además de 380 defunciones atribuibles a la TB, con una tasa de mortalidad de 5 por 100 000 habitantes ^12^. La mayor concentración de casos se observó en el Departamento Central y en Asunción, con 1500 casos (41,8% del total nacional). En relación con la coinfección TB/VIH la mayor proporción se registró en 2020 (8%), con una reducción a 6,8% en 2022 ^12^.

En América Latina y el Caribe, a pesar de las recomendaciones internacionales, la cobertura y el uso efectivo del tratamiento preventivo de la tuberculosis (TPT) en personas que viven con VIH siguen siendo subóptimos, con brechas entre elegibilidad y prescripción, así como variabilidad entre países y servicios de salud. Si bien, las actividades colaborativas TB/VIH han avanzado, persisten limitaciones en la integración de servicios y en la implementación sostenida del TPT, asociadas a barreras relacionadas con los profesionales de la salud, la organización de los servicios y la complejidad de los algoritmos clínicos ^13^.

En Sudamérica, y particularmente en Paraguay, la investigación en ciencia de la implementación aplicada a TB/VIH es limitada. La evidencia disponible se centra principalmente en aspectos epidemiológicos o resultados clínicos, con escasa evaluación de la fidelidad del TPT y de los determinantes contextuales y organizacionales que influyen en su implementación, y con una limitada utilización de marcos conceptuales formales de la ciencia de la implementación ^14^.

En este contexto, el presente estudio busca contribuir a cerrar esta brecha de conocimiento. El objetivo fue evaluar la adherencia autoinformada a los componentes esenciales del TPT, como estimación de fidelidad parcial de implementación, y explorar los factores moderadores que influyen en su implementación en dos servicios de referencia para personas que viven con VIH en el Departamento Central, Paraguay, 2023.

**MENSAJES CLAVE**

Motivación del estudio. Avanzar en la ciencia de la implementación en VIH y tuberculosis es clave para reducir la brecha entre evidencia y práctica, optimizando la adopción de intervenciones efectivas en contextos reales y mejorando resultados en poblaciones vulnerables.

Principales hallazgos. La fidelidad global del tratamiento preventivo de la tuberculosis (TPT) fue baja, con una brecha entre la adherencia al contenido (64,2%) y a la frecuencia (31,3%). La sensibilización/educación presentó el peor desempeño (30,0%). Las barreras se concentraron en el entorno interno (fragmentación TB/VIH, recursos limitados, falta de protocolos, deficiencias de comunicación y monitoreo) y, a nivel individual, en creencias erróneas y baja autoeficacia.

Implicancias en salud pública. La evidencia orienta estrategias para fortalecer la implementación del TPT y reducir la transmisión comunitaria. El presente estudio forma parte de la tesis: Duarte-Zoilan D. Fidelidad en la implementación del tratamiento preventivo de la tuberculosis en personas con infección por el virus de la inmunodeficiencia humana en centros de referencia del Departamento Central, Paraguay, 2023 [tesis de maestría en epidemiologia]. Medellin; Facultad Nacional de Salud Pública, Universidad de Antioquia; 2023.

## MATERIALES Y MÉTODOS

### Diseño del estudio

Se realizó un estudio observacional descriptivo con un diseño de métodos mixtos convergente paralelo, orientado a evaluar la fidelidad de implementación de una intervención preventiva en el contexto de la coinfección TB/VIH. Los componentes cuantitativo y cualitativo se recolectaron de manera concomitante entre enero y mayo de 2023, se analizaron por separado y se integraron en la fase de interpretación. El componente cuantitativo se orientó a cuantificar la adherencia autoinformada del personal médico a la guía nacional ^12^, mientras que el componente cualitativo permitió identificar factores moderadores que actúan como barreras o facilitadores de la implementación. La integración de ambos enfoques se utilizó para explicar las brechas de adherencia observadas y generar implicancias para fortalecer la implementación del tratamiento preventivo de la TB.

### Marco conceptual

El estudio se orientó por dos marcos conceptuales complementarios de la ciencia de la implementación. En primer lugar, se utilizó el Marco de Fidelidad de Implementación de Carroll *et al*. ^15^, que define la fidelidad como el grado en que una intervención se implementa conforme a lo prescrito. De acuerdo con este marco, se reconoce que la fidelidad de implementación puede verse influida por diversos factores moderadores. Para su identificación y análisis sistemático se empleó el Modelo Consolidado para la Investigación en Implementación (CFIR) ^16^, que permitió clasificar barreras y facilitadores en cinco dominios: características de la intervención, entorno interno, entorno externo, características de los individuos y proceso de implementación. La integración de ambos marcos permitió interpretar cómo los factores contextuales y organizacionales influyen en la fidelidad parcial observada en las dimensiones de contenido y frecuencia del TPT.

### Intervención

La intervención evaluada fue el TPT en personas que viven con VIH, conforme a la guía nacional vigente y al algoritmo clínico para la prevención de la TB ^12^. El TPT se conceptualizó como una intervención multicomponente y secuencial, cuya implementación está condicionada al descarte previo de TB activa. De acuerdo con el algoritmo nacional, en personas que viven con VIH y en contactos domiciliarios menores de cinco años no se requiere la realización de pruebas de infección tuberculosa latente (p. ej., prueba cutánea de tuberculina o pruebas de liberación de interferón gamma) para iniciar el TPT, siempre que se haya descartado TB activa. Este criterio fue considerado explícitamente en la definición operativa de la intervención y en la evaluación de la fidelidad parcial de implementación del TPT en el presente estudio ^12^.

### Modelo lógico

Se desarrolló un modelo lógico para describir la cadena causal que vincula los insumos del programa (capacitación, directrices normativas y sistemas de información), las actividades planificadas, los productos inmediatos y los resultados esperados a corto, mediano y largo plazo de la implementación del TPT (figura 1). Este modelo orientó la integración e interpretación de los hallazgos cuantitativos y cualitativos.

**Figura 1:** Modelo lógico para la implementación del tratamiento preventivo de la tuberculosis en personas que viven con VIH. Paraguay, 2023.

TB: tuberculosis; VIH: virus de la inmunodeficiencia humana; TPT: tratamiento preventivo para tuberculosis.

### Participantes, muestra y criterios de elegibilidad

El estudio se realizó en dos centros de referencia del Departamento Central, Paraguay, destinados a la atención de personas que viven con VIH, que funcionan como servicios de atención integral dentro de la red pública de salud bajo la responsabilidad del Ministerio de Salud Pública y Bienestar Social. Estos centros concentran aproximadamente el 75% de los pacientes en seguimiento. Adicionalmente, participaron gestores de los programas nacionales de TB y VIH.

En el componente cuantitativo, se invitó a participar al 100% de los médicos responsables de la atención de personas que viven con VIH en ambos centros.

En el componente cualitativo, los participantes fueron reclutados mediante muestreo intencional por criterio, e incluyeron cuatro gestores de programas (dos de TB y dos de VIH), dos médicos y cuatro usuarios de los servicios. Dado el carácter exploratorio del estudio y las restricciones operativas, no se buscó alcanzar saturación teórica formal.

### Fuentes de datos y operacionalización de variables

*Datos cuantitativos*

En este estudio, la fidelidad de implementación del TPT se conceptualizó como el grado de cumplimiento de los componentes esenciales de la guía nacional y fue operacionalizada de manera parcial, enfocándose en dos dimensiones: contenido (qué componentes del TPT se implementan) y frecuencia (con qué regularidad se implementan). Otras dimensiones del marco, como la cobertura poblacional, la dosis administrada y la calidad observada de la entrega, no fueron evaluadas. Estas dimensiones se midieron mediante el autorreporte del personal médico, utilizándose la adherencia a la guía nacional como un *proxy* operativo de fidelidad parcial de implementación.

Se aplicó un cuestionario estructurado que incluyó variables sociodemográficas y 40 ítems organizados en tres componentes del TPT: diagnóstico, consejería-concientización y manejo clínico. Cada componente incluyó ítems para la dimensión contenido (diagnóstico: 10; consejería-concientización: 3; manejo clínico: 9) y para la dimensión frecuencia de implementación (diagnóstico: 10; consejería-concientización: 3; manejo clínico: 5).

Las respuestas en escala tipo Likert fueron dicotomizadas a priori para definir adherencia. Para la dimensión de contenido, se consideraron respuestas adherentes las categorías «importante» y «muy importante»; para la frecuencia de implementación, las categorías «para todos los nuevos usuarios o anualmente para usuarios en seguimiento» y «siempre para todos los usuarios».

Para cada ítem, la adherencia se expresó como el porcentaje de participantes con respuestas clasificadas como adherentes. Posteriormente, se calculó un puntaje de adherencia por componente como el promedio simple de los porcentajes de sus ítems constitutivos. Finalmente, se estimó un puntaje global de adherencia como el promedio simple de los tres componentes evaluados, otorgando igual peso a cada uno. Este valor se interpretó como una estimación sintética de fidelidad parcial de implementación. Los niveles de adherencia (y, por extensión, de fidelidad parcial) se clasificaron como baja (0-50%), moderada (51-79%) y alta (80-100%) ^18^^-^^19^.

El cuestionario se adaptó de un instrumento previamente publicado y utilizado para evaluar la implementación de directrices de tamizaje de TB en clínicas de atención del VIH en Ghana ^17^. El instrumento fue sometido a traducción directa al español y a revisión por un panel de expertos con experiencia en TB, VIH y salud pública, con el fin de asegurar la equivalencia semántica, conceptual y contextual. Posteriormente, se realizaron adaptaciones culturales y operativas para alinearlo con las directrices nacionales de tuberculosis del Paraguay y con la organización local de los servicios TB/VIH. La validez de contenido se estableció mediante consenso cualitativo del panel de expertos y el instrumento fue sometido a una prueba piloto con un número reducido de profesionales, lo que permitió realizar ajustes menores en la redacción y comprensión de los ítems. La confiabilidad se evaluó mediante un análisis descriptivo de consistencia interna por dominios; sin embargo, debido al tamaño muestral reducido, no fue posible estimar coeficientes robustos de confiabilidad, como el alfa de Cronbach.

Se utilizaron dos escalas tipo Likert:

Importancia del contenido: «nada importante», «poco importante», «neutral», «importante» y «muy importante».

Frecuencia de implementación: «nunca», «raramente», «para algunos usuarios dependiendo de los síntomas», «para todos los nuevos usuarios o anualmente para usuarios en seguimiento» y «siempre para todos los usuarios».

*Datos cualitativos*

Se realizaron entrevistas semiestructuradas de carácter exploratorio, utilizando guías elaboradas específicamente para este estudio. Las guías se formularon a partir de los constructos del CFIR y de los moderadores del marco de fidelidad de implementación de Carroll, e incluyeron preguntas abiertas orientadas a explorar percepciones sobre la intervención, su complejidad, los procesos de implementación, las estrategias de facilitación y la capacidad de respuesta de los participantes. Se desarrollaron versiones diferenciadas de las guías según el tipo de actor (gestores, médicos y usuarios), manteniendo una estructura conceptual común y adaptando el lenguaje y los énfasis temáticos al rol de cada participante.

### Análisis de los datos

*Análisis cuantitativo*

Se realizó un análisis descriptivo de las variables cuantitativas del estudio, incluyendo las medidas de adherencia por ítem, las puntuaciones de adherencia por componente (diagnóstico, sensibilización/educación y manejo clínico) y la estimación global de fidelidad parcial de implementación. Para cada ítem, la adherencia se calculó como el porcentaje de respuestas clasificadas como adherentes respecto al total de respuestas posibles. En la escala tipo Likert, se consideraron respuestas adherentes: para la dimensión de contenido, las categorías «importante» y «muy importante»; y para la dimensión de frecuencia, «para todos los nuevos usuarios o anualmente para usuarios en seguimiento» y «siempre para todos los usuarios». Posteriormente, se estimaron porcentajes de adherencia por dimensiones (contenido y frecuencia) y por componentes, y finalmente se calculó un puntaje global de fidelidad parcial como la proporción de respuestas adherentes respecto al total de respuestas posibles en ambas dimensiones. Los resultados se presentaron con medianas y rangos intercuartílicos (RIC) y frecuencias absolutas y relativas. El procesamiento y análisis de los datos se realizó utilizando el software Epi Info versión 7.2.5.0.

*Análisis cualitativo*

Las entrevistas se grabaron en audio, se transcribieron de forma literal y se verificaron para asegurar su exactitud. Se realizó un análisis de contenido dirigido, organizando inicialmente las transcripciones en categorías vinculadas a la fidelidad de implementación y, posteriormente, aplicando una codificación deductiva basada en los constructos del CFIR para clasificar barreras y facilitadores en sus cinco dominios. El análisis se realizó de forma manual mediante matrices analíticas en Microsoft Excel. Para fortalecer la confiabilidad del análisis, la codificación y la interpretación de los hallazgos fueron revisadas y discutidas entre los investigadores hasta alcanzar consenso. Los resultados se integraron posteriormente mediante triangulación con los hallazgos cuantitativos en la fase de interpretación.

*Integración de métodos mixtos*

Los hallazgos cuantitativos y cualitativos se integraron mediante un enfoque de triangulación convergente, comparando las puntuaciones de fidelidad (contenido, frecuencia y medidas integradas por componente) con los resultados cualitativos basados en el CFIR para identificar convergencias, complementariedades o discrepancias. La interpretación fue orientada por el modelo lógico, destacando cómo los insumos del programa y los procesos clínicos influyeron en los patrones de fidelidad observados, y se elaboró una presentación conjunta para ilustrar los factores que explican las brechas de implementación.

### Prueba piloto

Se realizó una prueba piloto con dos médicos y dos pacientes para evaluar la claridad y comprensibilidad del cuestionario y de las guías de entrevista. Los datos del piloto no se incluyeron en el análisis final.

### Consideraciones éticas

Todos los datos fueron anonimizados y tratados de forma confidencial, de conformidad con las normativas paraguayas vigentes y los principios éticos de la Declaración de Helsinki. El estudio fue clasificado como de riesgo mínimo, ya que no implicó intervenciones clínicas ni modificación de la atención habitual. Todos los participantes otorgaron su consentimiento informado por escrito, y se les garantizó que su participación era voluntaria y que su decisión no tendría repercusiones en la atención recibida ni en su relación laboral o institucional. El estudio fue aprobado por el Comité de Ética en Investigación de la Facultad Nacional de Salud Pública de la Universidad de Antioquia (Registro n.º 21030002-00231-2022) y por la Dirección General de Vigilancia de la Salud del Ministerio de Salud Pública y Bienestar Social.

## RESULTADOS

En el componente cuantitativo participaron 11 médicos, mayoritariamente hombres (63,6%), con una mediana de edad de 35 años (RIC: 30-41 años) y una mediana de antigüedad en el cargo de 60 meses (RIC: 10-144). La mayoría se desempeñaba en hospitalización (45,4%) o en modalidad combinada hospitalización/consulta ambulatoria (36,4%) y residía en el Departamento Central (90,9%). El componente cualitativo incluyó 10 participantes: seis gestores y profesionales de salud (programas de TB y VIH y médicos), con una mediana de edad de 43,5 años (RIC: 42-49 años), y cuatro usuarios de los servicios, con una mediana de edad de 34,5 años (RIC: 25,5-47 años), distribución equitativa por sexo y niveles educativos variados (tabla 1).

**Tabla 1:** Características sociodemográficas de los participantes en la implementación del tratamiento preventivo de la tuberculosis en personas que viven con VIH. Paraguay, 2023.

Variable			n	%
Médicos-componente cuantitativo (n=11)				
	Género			
		Masculino	7	63,6
		Femenino	4	36,4
	Edad (años)^a^		35 (27-45)	
	Servicio donde labora			
		Hospitalización	5	45,4
		Consulta ambulatoria	2	18,2
		Ambos	4	36,4
	Departamento de procedencia			
		Central	10	90,9
		Guairá	1	9,1
	Ciudad de procedencia			
		Asunción	3	27,2
		Otras ciudades del Departamento Central	8	72,8
	Antigüedad en el cargo (meses)^a^		60 (10-144)	
Participantes del componente cualitativo (n=10)				
	Gestores y profesionales de la salud (n=6)			
	Edad (años)^a^		43,5 (39-55)	
	Género			
		Masculino	2	33,3
		Femenino	4	66,6
	Cargo			
		Programa TB	2	33,3
		Programa VIH	2	33,3
		Médico/a	2	33,3
	Usuarios de los servicios (n=4)			
	Edad (años)^a^		34,5 (24-52)	
	Género			
		Masculino	2	50,0
		Femenino	2	50,0
	Nivel educativo			
		Primario	1	25,0
		Secundario	1	25,0
		Universitario	2	50,0

TB: tuberculosis; VIH: virus de la inmunodeficiencia humana.

Los porcentajes se calcularon sobre el total de participantes de cada subgrupo.

a Mediana (rango intercuartílico).

En el componente 1 (diagnóstico de la TB) se observó una adherencia moderada al contenido, pero una baja frecuencia de implementación. Aunque la importancia atribuida a la evaluación de signos y síntomas alcanzó el 65,6%, la frecuencia de estas prácticas en la atención rutinaria fue del 34,4%. Además, una proporción relevante de médicos reportó preguntar rara vez sobre contacto con casos activos de TB y dificultades de acceso a pruebas diagnósticas. La adherencia integrada de este componente fue del 52,1%, correspondiente a un nivel moderado.

El componente 2 (sensibilización y educación) presentó los niveles más bajos de adherencia. Una proporción considerable de médicos asignó baja importancia a la indicación del TPT en personas que viven con VIH con antecedente de contacto con TB activa, y la frecuencia de implementación de actividades educativas fue escasa. La adherencia integrada de este componente fue del 30,3%, reflejando un déficit sustancial en la ejecución de acciones educativas esenciales.

En el componente 3 (manejo clínico), si bien la mayoría de los médicos reconoció la importancia del acceso a algoritmos clínicos, se evidenció una brecha entre el conocimiento de las directrices y su aplicación práctica. La valoración del contenido clínico fue moderada (67,7%), mientras que la frecuencia de implementación fue baja (33,0%), particularmente en la indicación del TPT a personas asintomáticas o con resultados negativos para TB pulmonar. La adherencia integrada de este componente alcanzó el 55,1%, correspondiente a un nivel moderado (figura 2). Al integrar los tres componentes, el nivel global de fidelidad parcial de implementación del TPT -basado en las dimensiones de contenido y frecuencia- fue del 50,0%, lo que indica un bajo nivel de cumplimiento operativo de las directrices nacionales (tabla 2).

**Figura 2:** Adherencia de los médicos en la implementación del tratamiento preventivo de la tuberculosis en personas que viven con VIH según dimensiones y componentes. Paraguay, 2023.

^a^ Frecuencia. 1: nunca, 2: raramente, 3: para algunos usuarios dependiendo de los síntomas, 4: para todos los nuevos usuarios o anualmente para usuarios en seguimiento y 5: siempre para todos los usuarios. ^b^ Contenido.1: nada importante, 2: poco importante, 3: neutral, 4: importante y 5: muy importante.

**Tabla 2:** Fidelidad de los médicos en la implementación del tratamiento preventivo de la tuberculosis en personas que viven con VIH según dimensiones y componentes. Paraguay, 2023.

Resultado de implementación	Dimensiones	Componentes	Respuestas en las categorías esperadas^a^	Nivel alcanzado^b^	%
			Respuestas/ respuestas posibles		
Adherencia	Contenido	Diagnóstico de la TB	84/128	Moderado	65,6
		Sensibilización de la TB	16/33	Bajo	46,5
		Manejo clínico de la TB	67/99	Moderado	67,7
	Frecuencia	Puntaje total	167/260	Moderado	64,2
		Diagnóstico de la TB	40/110	Bajo	34,4
		Sensibilización de la TB	4/33	Bajo	12,1
		Manejo clínico de la TB	18/55	Bajo	33
		Puntaje total	62/198	Bajo	31,3
Fidelidad global	Contenido y frecuencia integrados	Diagnóstico de la TB	124/238	Moderado	52,1
		Sensibilización de la TB	20/66	Bajo	30,3
		Manejo clínico de la TB	85/154	Moderado	55,1
		Puntaje total	229/458	Bajo	50

TB: tuberculosis.

a Respuestas en las categorías esperadas corresponden a las respuestas consideradas adherentes para cada dimensión.

b Los niveles de fidelidad se clasificaron como baja (0-50%), moderada (51-79%) y alta (80-100%).

Durante la fase cualitativa se realizaron entrevistas semiestructuradas con cuatro gestores de programas, dos médicos de los Servicios de Atención Integral y cuatro usuarios, quienes aportaron perspectivas complementarias sobre la gestión programática, la atención clínica y la experiencia del tratamiento preventivo de la TB. El análisis cualitativo permitió identificar barreras y facilitadores organizados en los cinco dominios del CFIR. Los hallazgos mostraron que factores organizacionales, individuales y contextuales influyen en la prescripción, el inicio y la continuidad del tratamiento preventivo en personas que viven con VIH.

Las principales barreras se concentraron en el entorno interno, destacándose la fragmentación de los servicios TB/VIH, la limitada disponibilidad de recursos organizacionales, las brechas de conocimiento y creencias entre los profesionales de la salud, y la ausencia de procesos sistemáticos de planificación y retroalimentación. Entre los facilitadores, se identificaron la disposición del personal para recibir capacitación, el interés de algunos usuarios en acceder a información sobre el tratamiento preventivo y la alineación de las políticas nacionales con las recomendaciones internacionales. La síntesis de los constructos del CFIR, junto con la evidencia cualitativa y su interpretación analítica, se presenta en la tabla 3.

**Tabla 3:** Potenciales moderadores de la fidelidad de implementación del tratamiento preventivo de la tuberculosis en personas que viven con VIH según dominios del CFIR. Paraguay, 2023.

Constructo^a^		Evidencia (Fragmentos verbatim del estudio)^b^	Interpretación (Cómo influye en la fidelidad)
Dominio 1. Intervención			
	Complejidad de la intervención	«Los programas son completamente independientes en su gestión… la comunicación entre los servicios es dificultada…»; «Integración limitada de la atención tuberculosis/virus de la inmunodeficiencia humana…»	La intervención requiere coordinación en múltiples niveles (TB/VIH), lo que aumenta su complejidad y reduce la fidelidad en el diagnóstico, la prescripción y el seguimiento.
	Calidad y solidez de la evidencia	«Existen dudas generalizadas sobre la efectividad del tratamiento preventivo…»; «Algunos médicos creen que el tratamiento preventivo puede causar resistencia…»	La evidencia percibida como insuficiente genera baja confianza en el tratamiento preventivo, lo que reduce su adopción y fidelidad.
	Adaptabilidad	«El seguimiento en personas que viven con virus de la inmunodeficiencia humana no es complejo; el tratamiento preventivo solo necesita ser especificado y volverse más claro…»	Existen oportunidades de adaptación local; sin embargo, los componentes esenciales no están claramente definidos.
	Claridad del diseño	«La directriz nacional no especifica para quién está indicado el tratamiento preventivo… no está claro…»	La falta de claridad en las directrices clínicas reduce la estandarización y aumenta la variabilidad de la práctica.
Dominio 2. Entorno externo			
	Necesidades y recursos de los pacientes	«Esta es realmente la primera vez que escucho… nunca había oído que existiera un tratamiento para prevenir la tuberculosis…»	Los pacientes no perciben la tuberculosis como un riesgo prioritario, lo que genera una demanda pasiva y menor presión para la prescripción del tratamiento preventivo.
	Redes interorganizacionales externas	«No existe interconexión entre los sistemas de información de ambos programas…»	Los sistemas de tuberculosis/virus de la inmunodeficiencia humana fragmentados dificultan el seguimiento clínico continuo y comprometen la completitud de la implementación.
	Políticas e incentivos externos	Directrices nacionales alineadas con la Organización Mundial de la Salud (observaciones de campo).	Las políticas nacionales son favorables; sin embargo, la implementación local es frágil debido a la baja articulación operativa.
Dominio 3. Entorno interno			
	Estructura organizacional	«La coordinación entre los niveles regional, distrital y local es limitada…»; «El centro de referencia en VIH no cuenta con un programa de tuberculosis…»	La estructura organizacional fragmentada afecta la adecuada transferencia del protocolo de tratamiento preventivo y reduce la coherencia organizacional.
	Recursos disponibles	«Existen vacíos en el registro de la información…»; «Falta de documentos normativos claros…»	Recursos técnicos, informacionales y de apoyo insuficientes constituyen una barrera para la implementación consistente.
	Clima de implementación	«Se observó que la búsqueda activa de casos de tuberculosis no fue totalmente implementada…»	El clima organizacional no es favorable; el tratamiento preventivo no se prioriza entre las actividades rutinarias.
	Redes de comunicación y coordinación	«La falta de comunicación dificulta el trabajo colaborativo…»	La comunicación interprogramática deficiente reduce la calidad de la implementación.
Dominio 4. Características de los individuos			
	Conocimientos y creencias sobre la intervención	«Muchos colegas creen que el tratamiento preventivo es solo para niños…»	Creencias incorrectas constituyen una barrera crítica para la adherencia en el diagnóstico y el manejo clínico.
	Autoeficacia	Dudas sobre resistencia, complejidad y falta de capacitación («necesitamos más información…»).	La baja confianza reduce la probabilidad de prescripción del tratamiento preventivo y el uso del algoritmo clínico.
	Etapa de cambio / preparación individual	Interés en capacitación (hallazgos del diario de campo).	Existe disposición para la mejora si se proporcionan herramientas adecuadas.
	Necesidades de los pacientes	«Había escuchado una vez… pero nunca pregunté y el médico no me explicó nada…»	El bajo nivel de conocimiento de los pacientes limita la adhesión y aceptación, reduciendo la presión para la implementación.
Dominio 5. Proceso de implementación			
	Planificación	Ausencia de protocolos y flujogramas claros específicos para el tratamiento preventivo en adultos.	La falta de planificación detallada genera inconsistencias en la prescripción y el seguimiento.
	Ejecución	«La búsqueda activa de casos de tuberculosis no es monitoreada…»	Ejecución incompleta de los componentes esenciales del tratamiento preventivo.
	Participación de las partes interesadas	Usuarios: «solicitando información para recibir tratamiento preventivo…»; programas nacionales dispuestos a ofrecer capacitación.	Aunque existen actores motivados, falta un mecanismo articulado de implementación.
	Reflexión y evaluación (retroalimentación)	No existe evidencia de monitoreo interno regular.	La ausencia de retroalimentación estructurada impide la mejora continua y reduce la fidelidad.

TB: tuberculosis; VIH: virus de la inmunodeficiencia humana; TPT: tratamiento preventivo de la tuberculosis.

a Constructos del Modelo Consolidado para la Investigación en Implementación (CFIR).

b Fragmentos textuales anonimizados de entrevistas y observaciones de campo.

La integración de los hallazgos cuantitativos y cualitativos mostró concordancia entre los componentes con menor fidelidad de implementación y los dominios que concentraron las principales barreras. En particular, las debilidades en los procesos organizacionales y en la coordinación entre los programas de TB y VIH ayudan a explicar la baja frecuencia de implementación observada en los componentes evaluados. Un resumen de esta triangulación se presenta en la tabla 4, donde se relacionan los resultados cuantitativos, las barreras cualitativas y su interpretación integrada.

**Tabla 4:** Triangulación de los hallazgos cuantitativos y cualitativos mediante modelo lógico en la implementación del tratamiento preventivo de la tuberculosis en personas que viven con VIH. Paraguay, 2023.

Hallazgo cuantitativo	Hallazgo cualitativo	Interpretación conjunta (modelo lógico)^a^
Baja frecuencia de actividades clínicas clave (diagnóstico, educación y manejo clínico).	Entorno interno: escasez de recursos, ausencia de protocolos claros, inexistencia de supervisión y retroalimentación.	Los procesos clínicos del tratamiento preventivo no se ejecutan plenamente debido a debilidades en los insumos y en los procesos organizacionales.
La educación en salud presentó el menor desempeño (12,1% de conformidad).	Individuos: concepciones erróneas del equipo, baja autoeficacia; usuarios con conocimiento limitado sobre el tratamiento preventivo de la tuberculosis.	La educación en salud insuficiente y las percepciones incorrectas sobre el tratamiento preventivo influyen negativamente en la adhesión a este componente.
Baja adherencia global (50,0%).	Intervención y entorno interno: complejidad TB/VIH, fragmentación organizacional y comunicación limitada.	La integración deficiente entre los programas de tuberculosis y VIH interrumpe los flujos clínicos y reduce la fidelidad global.
Brechas entre la importancia percibida y el desempeño real.	Proceso: ausencia de planificación, monitoreo y retroalimentación estructurada.	Aunque el equipo conoce las directrices, los procesos no están estructurados para asegurar una implementación sostenida.

TB: tuberculosis, VIH: virus de la inmunodeficiencia humana, TPT: tratamiento preventivo de la tuberculosis.

a Interpretación basada en el modelo lógico de implementación que integra los resultados cuantitativos y cualitativos.

## DISCUSIÓN

El presente estudio evaluó la fidelidad parcial de implementación del TPT en personas que viven con VIH en dos centros de referencia del Paraguay, desde un enfoque de ciencia de la implementación. El principal hallazgo fue un bajo nivel global de fidelidad parcial (50%), caracterizado por una brecha entre la adherencia al contenido (64,2%) y la frecuencia de implementación (31,3%). Este patrón sugiere que, aunque los profesionales reconocen la importancia de las recomendaciones clínicas, su aplicación sistemática en la práctica asistencial es limitada.

Estos resultados son coherentes con la literatura de ciencia de la implementación, que señala que el conocimiento de las directrices, por sí solo, es insuficiente para garantizar una implementación fiel, especialmente en intervenciones complejas y multicomponentes desarrolladas en sistemas de salud fragmentados ^14^^,^^16^^,^^20^. En particular, el componente de sensibilización y educación presentó los niveles más bajos de desempeño, con la menor frecuencia de implementación (12,1%) y adhesión integrada (30,3%), lo que resulta preocupante dado el rol central de la educación del paciente para favorecer la aceptación, adherencia y continuidad del TPT ^21^.

Los hallazgos cualitativos permitieron explicar estos patrones cuantitativos. Desde la perspectiva del Modelo Consolidado para la Investigación en Implementación, se identificaron barreras en los cinco dominios, destacándose el entorno interno como el principal foco de obstáculos. La fragmentación organizacional entre los programas de TB y VIH, las limitaciones en los sistemas de información, la debilidad de los canales de comunicación y la ausencia de directrices operativas claras restringieron la oferta consistente del TPT. Estos resultados concuerdan con estudios realizados en otros contextos de ingresos bajos y medios, donde la integración limitada de los servicios TB/VIH compromete la efectividad de las intervenciones preventivas ^22^^-^^23^.

La complejidad de la intervención emergió como un factor clave. Aunque las directrices nacionales e internacionales recomiendan el TPT en personas que viven con VIH tras descartar TB activa, sin requerir pruebas diagnósticas adicionales ^1^^,^^12^, una proporción importante de médicos atribuyó baja relevancia a su indicación en adultos asintomáticos. Este hallazgo refleja cómo la ambigüedad percibida y la interpretación heterogénea de las recomendaciones clínicas pueden debilitar la fidelidad de la implementación, aun cuando las políticas estén alineadas con las recomendaciones de la OMS ^4^^,^^7^. Resultados similares se han descrito en estudios de Ghana y Perú, donde la incertidumbre sobre criterios de elegibilidad y las preocupaciones por resistencia farmacológica redujeron la adherencia de los profesionales al TPT ^17^^,^^23^.

Asimismo, factores relacionados con las características de los individuos influyeron negativamente en la fidelidad. Creencias erróneas sobre el TPT, junto con una menor autoeficacia percibida, afectaron la toma de decisiones clínicas y la oferta de educación asociada. Es necesario un seguimiento estricto por parte de los profesionales sanitarios para garantizar la adherencia al tratamiento en personas con TB/VIH y mejorar las tasas de éxito terapéutico, considerando que la evidencia previa indica que la confianza y la competencia percibida de los profesionales constituyen determinantes clave para el éxito de la implementación, especialmente en intervenciones preventivas cuyos beneficios no son inmediatos ^24^.

Finalmente, la integración de los hallazgos cuantitativos y cualitativos mediante el modelo lógico y el Marco conceptual de Fidelidad, permitió esclarecer cómo las deficiencias en los insumos (capacitación, claridad normativa y sistemas de información) y en los procesos (tamizaje, educación, prescripción, documentación y seguimiento) se tradujeron en una baja frecuencia de implementación. Desde una perspectiva programática, estos resultados sugieren que mejorar la fidelidad del TPT en Paraguay requiere ir más allá de la difusión de directrices y avanzar hacia estrategias activas de facilitación, como algoritmos clínicos simplificados, capacitación continua, fortalecimiento de la documentación y mecanismos sistemáticos de auditoría y retroalimentación, que han demostrado mejorar la implementación en otros contextos ^25^^-^^26^.

Este estudio presenta algunas limitaciones. La validación del instrumento se basó principalmente en revisión por expertos y adaptación contextual, sin estimación de métricas cuantitativas formales de validez o confiabilidad, lo que constituye una limitación metodológica. En segundo lugar, la fidelidad de implementación fue evaluada de manera parcial, limitada a las dimensiones de contenido y frecuencia y basada en autorreporte del personal médico, lo que puede haber introducido sesgos de recuerdo y deseabilidad social. Además, no fue posible evaluar otros componentes centrales de la fidelidad, como la cobertura poblacional, la dosis administrada o la calidad de la entrega, debido a la disponibilidad limitada de registros clínicos y restricciones de acceso, lo que impidió la triangulación con medidas objetivas.

Asimismo, el componente cualitativo fue exploratorio, con un tamaño muestral reducido y sin búsqueda de saturación teórica formal, por lo que sus hallazgos deben interpretarse como indicativos y no generalizables. El estudio se realizó en dos servicios de referencia especializados, por lo que los resultados son principalmente aplicables a contextos similares de atención en VIH y no necesariamente representativos del nivel nacional. Finalmente, aunque el uso de métodos mixtos fortaleció la interpretación de los hallazgos, el diseño observacional y transversal del estudio no permite establecer relaciones causales ni evaluar cambios longitudinales en la implementación del TPT.

En conclusión, la adherencia autoinformada a los componentes esenciales del tratamiento preventivo de la TB fue baja, reflejando una fidelidad parcial limitada en las dimensiones de contenido y frecuencia. Los factores moderadores identificados se concentraron principalmente en el entorno organizacional y en características individuales de los profesionales de salud, lo que ayuda a explicar las brechas observadas entre la importancia atribuida a las directrices y su aplicación en la práctica clínica. Estos hallazgos sugieren que fortalecer la facilitación de la implementación, mediante algoritmos claros, capacitación continua, integración de los servicios TB/VIH y mecanismos sistemáticos de retroalimentación, podría mejorar la implementación del TPT en personas que viven con VIH.
